# The predictive value of lymphocyte to monocyte ratio for overall survival in cholangiocarcinoma patients with hepatic resection

**DOI:** 10.1002/cam4.5712

**Published:** 2023-02-24

**Authors:** Minghe Lv, Kun Wang, Zhiyuan Zhang, Zhen Zhang, Juefeng Wan

**Affiliations:** ^1^ Department of Radiation Oncology, Fudan University Shanghai Cancer Center Fudan University Shanghai China; ^2^ Department of Oncology Shanghai Medical College Fudan University Shanghai China; ^3^ Shanghai Clinical Research Center for Radiotherapy Oncology Shanghai Key Laboratory of Radiation Oncology Shanghai China

**Keywords:** cholangiocarcinoma, LMR, lymphocyte, monocyte, overall survival

## Abstract

**Background:**

There is considerable heterogeneity in clinical behavior and survival outcomes in patients with cholangiocarcinoma (CCA), and the prognosis of CCA patients is poor. We proposed lymphocyte to monocyte ratio (LMR) as a novel prognostic element for CCA patients with hepatic resection in present study.

**Methods:**

By retrospectively analyzing the clinical data of 145 CCA patients with hepatic resection, we determined the optimal LMR cutoff value according to the receiver operating characteristic (ROC). We comparatively analyzed the clinical features of CAA patients between low LMR group and high LMR group, mainly including overall survival (OS) analysis by using the Kaplan‐Meier method, univariate and multivariate Cox regression.

**Results:**

We found there was a longer OS in CCA patients of the high LMR group than the low LMR group. The total median OS of cholangiocarcinoma patients were 13.6 months, and the OS of low LMR group was markedly lower than the high LMR group. The 1‐year, 3‐year, and 5‐year OS of high LMR group were respectively 62.9%, 32.4%, and 16.4%, and were significantly higher the cholangiocarcinoma patients of low LMR group (40.2%, 16.4%, and 0%). Multivariate regression analyses showed that preoperative cholangitis, elevated CEA level and nerve invasion were risk factors for the OS of cholangiocarcinoma patients, while the high LMR level and postoperative treatment were protective factors for the OS of cholangiocarcinoma patients.

**Conclusions:**

Preoperative LMR was a vital prognostic factor to predict the prognosis of CCA patients with hepatic resection and provided additional prognostic value beyond standard clinicopathological parameters.

## Graphical abstract

1.By retrospectively analyzing the clinical data of 145 CCA patients with hepatic resection, we determined the optimal LMR cutoff value according to the receiver operating characteristic (ROC).

2.We found there was a longer OS in CCA patients of the high LMR group than the low LMR group.

3.Preoperative LMR was a vital prognostic factor to predict the prognosis of CCA patients with hepatic resection and provided additional prognostic value beyond standard clinicopathological parameters.

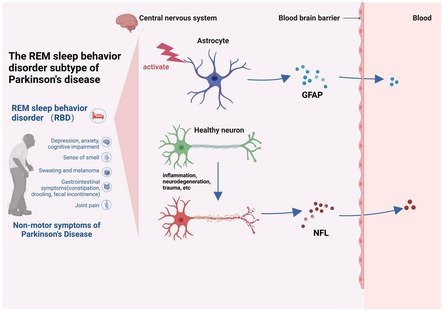

## 1 INTRODUCTION

Cholangiocarcinoma (CCA), including intrahepatic and extrahepatic cholangiocarcinoma, is a kind of epithelial malignant tumor originating in the biliary tract and is a highly invasive biliary tract tumor with poor survival.^1^ Overall, CCA constitutes 3% of all gastrointestinal tumors, representing the second most frequently diagnosed primary liver cancer following hepatocellular carcinoma (HCC)^2^ and the overall incidence of CCA has been increasing in countries around the world,^3^, ^4^ and the 5‐year overall survival (OS) rate of CAA was <5%. Surgery and liver transplantation may be potential treatments for patients with CCA. The current study does not recommend liver transplantation as a first choice because of its technical difficulty and high cost. Despite the changes in surgical methods and techniques, the resection rate of patients with cholangiocarcinoma is still very low. Postoperative recurrence and metastasis are the main causes of poor prognosis. In addition, the discovery of effective blood prognostic signatures for recurrence and survival in CCA patients after hepatectomy remains an unmet need.

Recent studies have shown that inflammation is considered to be a hallmark feature of cancer development and progression, and that proinflammatory cytokines and chemokines in the cancer microenvironment lead to the survival and proliferation of tumor cells, metastasis, angiogenesis, and destruction of adaptive immunity, thereby affecting survival and prognosis.^5^ On the one hand, chronic inflammation triggers the local accumulation of monocytes, platelets, and neutrophils, secreting cytokines and inflammatory factors to induce tumor angiogenesis and metastasis. On the other hand, the increase of monocytes and lymphocytes will enhance the ability to resist tumor invasion.^6^ The impact of inflammatory‐based scores on cancer therapy outcomes has been extensively studied. Previous studies found that there was a prognostic value for inflammation biomarkers such as the radio among neutrophils, lymphocytes, C‐reactive protein (CRP), and platelet.^7^, ^8^, ^9^ Previous studies have shown that intrahepatic cholangiocarcinoma patients with a low level of NLR (neutrophil to lymphocyte ratio) and PLR (platelet to lymphocyte ratio) had longer OS than those with high levels, suggesting that low levels of NLR and PLR were associated with poorer prognosis.^9^ Lymphocyte to monocyte ratio (LMR), a relatively new inflammatory‐related score and a biomarker associated with translational inflammatory response, has been exhibited to have potential prognostic value in patients with kinds of tumors, such as lymphoma,^10^ colorectal cancer,^11^ and lung cancer.^12^ Known risk factors for CCA, such as liver fluke, liver calculus, and primary sclerosing cholangitis, can cause chronic inflammation of the liver, suggesting that inflammation is closely related to the carcinogenesis of CCA.^13^, ^14^ Furthermore, Studies showed that preoperative LMR is a very significant indicator that can be used to indicate whether patients with cholangiocarcinoma are resectable, and dynamic changes of LMR can accurately predict early recurrence in patients with advanced curable cholangiocarcinoma.^15^ However, the prognostic and survival significance of preoperative LMR for patients with postoperative CCA has not been reported in detail.

In this study, we aimed to assess the prognostic value of preoperative LMR in patients with CCA following hepatic resection and analyze risk factors for the overall survival of CCA patients with hepatic resection via using receiver‐operating characteristic (ROC) curve and establishing Cox regression analysis model.

## 2 MATERIALS AND METHODS

### 2.1 Patients

This study retrospectively analyzed 145 patients with newly diagnosed cholangiocarcinoma who underwent hepatectomy and received treatment at Fudan University Shanghai Cancer Center from August 2008 to October 2020. All patients underwent preoperative blood routine, liver and kidney function, and other indicators, as well as lung function, electrocardiogram, chest CT, and other routine items. Intraoperative ultrasound was performed to assess the likelihood of tumor load, residual liver, and negative margins. Anatomic excision is the preferred surgical method. Postoperative adjuvant therapy, including chemoradiotherapy, target area therapy, or immunotherapy, was determined according to the postoperative pathology or risk factors of the patient. This study was approved by the Medical Ethics Committee of Fudan University Shanghai Cancer Center. Patients included in this study met the following criteria: (1) pathologically and immunohistochemically confirmed diagnosis of cholangiocarcinoma, (2) received hepatic resection, (3) complete clinicopathological and follow‐up data. Patients were excluded from the study if they had mixed cancer, unknown tumor origin, or distant metastasis before surgery. The study was approved by the ethics committee of the hospital, and the ethical code is 2003215‐1.

### 2.2 Follow‐up and data extraction

The time of follow‐up ended in October 2021. All patients received informed consent. Overall survival (OS) was defined as the time interval from resection to death or the date of the last follow‐up. For each patient included in our analysis, we collected the following data: age, sex, HBV infection, the preoperative levels of carbohydrate antigen 19–9 (CA199), alpha fetal protein (AFP), and carcino‐embryonic antigen (CEA), TNM stage, vascular invasion, lymphatic metastasis, nerve invasion, bile duct infringement, tumor diameter, tumor number, satellite focal, pathological differentiation, tumor location, preoperative treatment, and postoperative treatment. We obtained absolute lymphocyte counts and absolute monocyte counts (AMC) in peripheral blood via the standard automated complete blood counts within 3 days before the surgical resection. The LMR was calculated through ALC divided by AMC.

### 2.3 Statistical analysis

The chi‐square test or Fisher's exact test was used to compare classification characteristics and ratios. The optimal cut‐off value for the LMR was determined using the maximum Yoden index (the point closest to the upper left of the ROC curve). The Kaplan–Meier method was used to calculate the survival probability, and the log‐rank test was used to compare the differences between groups. Cox proportional risk regression model was used for univariate and multivariate analysis. *p* < 0.05 was statistically significant. All statistical analyses were performed using the Social Science Program Statistical Software Package (SPSS Inc. Chicago, IL, Windows version 22.0). We use the survival, RMS, and GGplot2 packages in R (version 4.1.3) to generate a nomogram of multivariate analysis results.

## 3 RESULTS

### 3.1 Kaplan–Meier analysis curves for OS and ROC curve analysis of the LMR in cholangiocarcinoma patients

**FIGURE 1:**
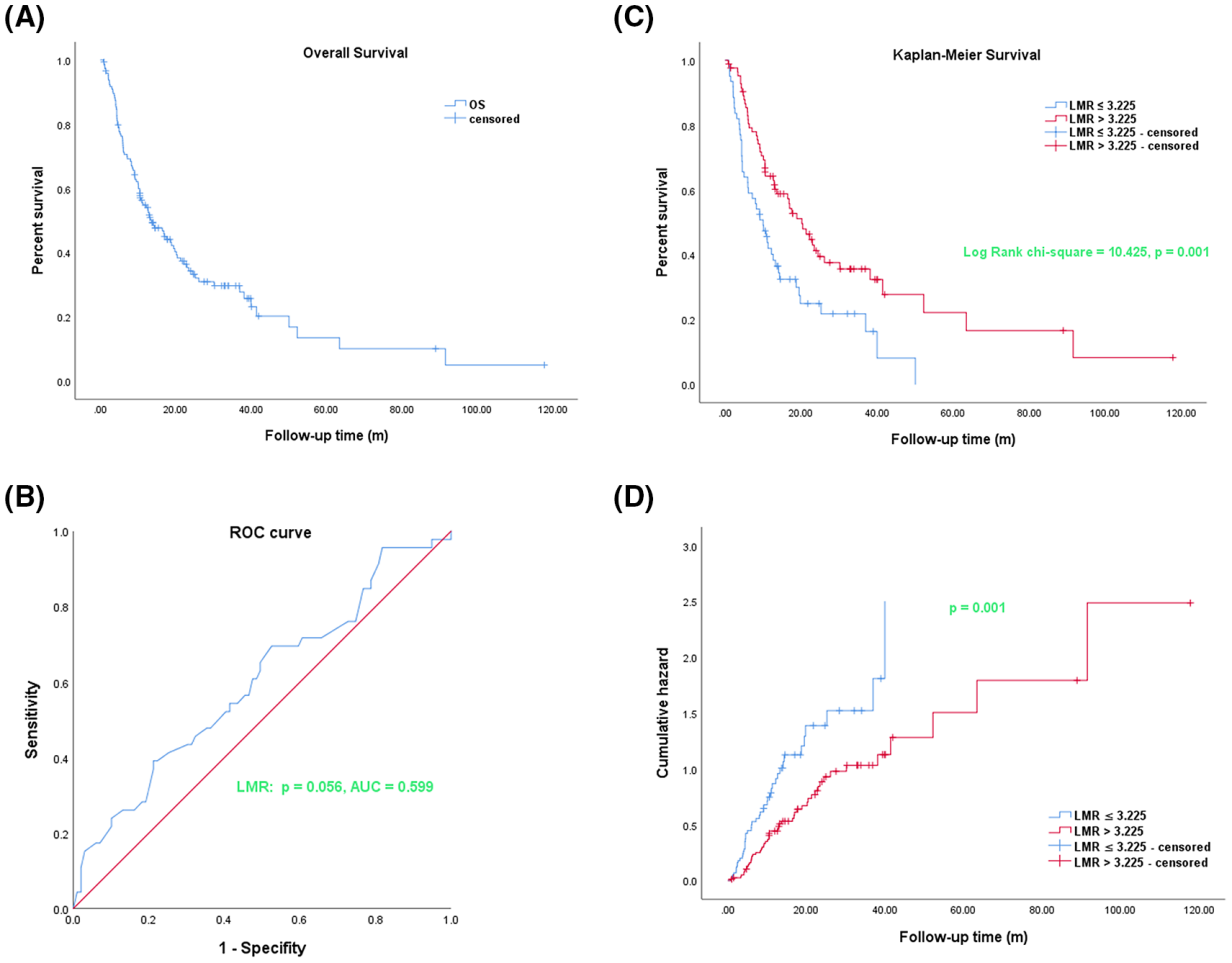
Kaplan–Meier analysis curves for overall survival and Receiver‐operating characteristic curve (ROC) curve analysis of the lymphocyte to monocyte ratio (LMR) in cholangiocarcinoma patients. (A) The Kaplan–Meier plot of overall survival (OS) of 145 cholangiocarcinoma patients. (B) ROC was used to determine the LMR cut‐off value for cholangiocarcinoma patients. (C) OS analysis of cholangiocarcinoma patients in low LMR (LMR ≤3.225) and high LMR (LMR >3.225) groups. (D) Survival risk analysis for overall survival of cholangiocarcinoma patients in low LMR (LMR ≤3.225) and high LMR (LMR >3.225) groups. *p* < 0.05 was statistically significant.

In this part, we respectively analyzed the OSof 145 cholangiocarcinoma patients who were newly treated and underwent surgical treatment at the Fudan University Shanghai Cancer Center from August 2008 to October 2020. As shown in Figure 1A, we used the Kaplan–Meier method to analyze the OS of 145 cholangiocarcinoma patients. Furthermore, we used the ROC curve to explore the diagnosis value and the optimum truncation value of preoperative LMR. The results as shown in Figure 1B, indicated that the optimal cut‐off value of LMR was 3.225, and the area under the curve (AUC) was 0.599 (95% CI 0.498–0.700), *p* = 0.056. We so divided these patients into two groups according to the optimal cut‐off of LMR: the low LMR group (LMR≤3.225) and high LMR group (LMR >3.225). The results of OS via the Kaplan–Meier analysis in the two groups showed that there was a longer OS in cholangiocarcinoma patients of the high LMR (LMR >3.225) group than the low LMR group (LMR≤3.225) (long‐rank chi‐square = 10.425, *p* = 0.001) **(**Figure 1C **)**. The results of the risk analysis function showed that the patients in the low LMR group exhibited a high risk, while the patients in the high LMR group exhibited a significantly low risk, and the *p* value was statistical significance after the comparison between the two groups **(**Figure 1D **)**.

### 3.2 The clinical characteristics and survival analysis results of cholangiocarcinoma patients in low and high LMR groups

A total of 145 newly diagnosed cholangiocarcinoma patients, with median age, was 60 years (range 26–80 years). Male accounted for 53.8% and female accounted for 46.2%. Among 145 patients, 42 (29%) were positive for HBV infection, and 103 (71%) were negative for HBV infection. The levels of CA199 of 35 (24.1%) patients were normal, while 110 (75.9%) patients were abnormal. The AFP levels of 19 (13.1%) patients and the CEA levels of 61 (42.1%) patients were elevated. 56 (38.6%) patients were diagnosed as I or II stage according to the eighth edition TNM stage, while 89 (61.4%) patients were diagnosed as III or IV stage. Among 145 patients, 50 (34.5%) had preoperative cholangitis and the ALB levels of 6 (4.1%) patients were decreased. The ALT levels of 33 (22.8%) patients, the AST levels of 38 (26.2%) patients, and the TBIL levels of 13 (9%) patients were elevated. 145 eligible patients were categorized into the low LMR and high LMR groups according to the optimal cut‐off value of the preoperative LMR. We further employed the chi‐square test to analyze the correlation between LMR and clinical characteristics, and the results showed that the level of CA199 and CEA, TNM stage, and tumor number as statistically significant factors related to the preoperative LMR of cholangiocarcinoma patients **(**Table 1 **)**. We also found that the total median OS of cholangiocarcinoma patients was 13.6 months (95% CI 8.91–18.29), the OS of the low LMR group (10 months, 95% CI 6.766–13.234) was markedly lower (*p* = 0.001) than the high LMR group (20.23 months, 95% CI 14.706–25.754). The median follow‐up time of 145 cholangiocarcinoma patients was 11.9 months (range 0.83–117.80 months). We found that as shown in Table 2, the 1‐year, 3‐year, and 5‐year total OS of 145 CCA patients were, respectively, 54.1%, 27.7%, and 10.1%. The 1‐year, 3‐year, and 5‐year OS in the low LMR group were, respectively, 40.2%, 16.4%, and 0%. The 1‐year, 3‐year, and 5‐year OS in the high LMR group were, respectively, 62.9%, 32.4%, and 16.4%, and were significantly higher than the OS of CCA patients of the low LMR group. Therefore, we concluded that LMR could serve as a novel and predictive index for the OS of cholangiocarcinoma patients.

**TABLE 1:** Clinical characteristics of 145 cholangiocarcinoma patients.

Characteristic	Number	Low LMR group	High LMR group	Chi‐square	*p*‐Value
		LMR≤ 3.225 (*n* = 61)	LMR >3.225 (*n* = 84)		
Age				1.21	0.271
≤55	48	23	25		
≥55	97	38	59		
Sex				0.075	0.784
Male	78	32	46		
Female	67	29	38		
HBV infection				0.383	0.536
No	103	45	58		
Yes	42	16	26		
CA199 (ng/ml)				5.064	**0.024**
<27	35	9	26		
≥27	110	52	58		
AFP (ng/mL)				<0.0001	0.997
<10	126	53	73		
≥10	19	8	11		
CEA (ng/mL)				8.072	**0.004**
<5.2	84	27	57		
≥5.2	61	34	27		
TNM stage				18.828	**<0.0001**
I + II	56	11	45		
III + IV	89	50	39		
Vascular invasion				2.841	0.092
No	55	28	27		
Yes	90	33	57		
Lymphatic metastasis				1.914	0.166
No	116	42	74		
Yes	39	19	20		
Nerve invasion				2.463	0.117
No	94	44	50		
Yes	51	17	34		
Bile duct infringement				1.071	0.301
No	124	50	74		
Yes	21	11	10		
Tumor diameter (cm)				1.657	0.198
≤2	22	12	10		
>2	123	49	74		
Tumor number				7.677	**0.006**
≤1	127	48	79		
>1	18	13	5		
Satellite focal				0.436	0.509
No	120	49	71		
Yes	25	12	13		
Preoperative cholangitis				2.039	0.153
No	95	44	51		
Yes	50	17	33		
Pathological type				0.1	0.751
ICC	89	36	53		
EHCC	56	25	31		
ALT				1.564	0.211
≤50	112	44	68		
>50	33	17	16		
AST				1.329	0.249
≤40	107	42	65		
>40	38	19	19		
ALB				8.619	**0.003**
≤35	6	6	0		
>35	139	55	84		
TBIL				0.076	0.782
≤26	132	56	76		
>26	13	5	8		

Abbreviations: ALB, albumin; AFP, alpha fetal protein; LMR, lymphocyte to monocyte ratio; CA199, carbohydrate antigen 19–9; CEA, carcino‐embryonic antigen; EHCC, extrahepatic cholangiocarcinoma; ICC, intrahepatic; cholangiocarcinoma; TBIL, total bilirubin.

Bold indicates significance level at *p* < 0.05.

**TABLE 2:** Basic clinical characteristics of survival analysis of cholangiocarcinoma patients in low and high LMR groups.

Group	Median survival				*p*‐value	Overall survival (%)		
	Estimated value	Standard	95% Confidence interval			1‐year	3‐year	5‐year
		error	Inferior limit	Upper limit				
LMR ≤3.225	10	1.65	6.766	13.234	**0.001**	40.2	16.4	0
LMR >3.225	20.23	2.818	14.706	25.754		62.9	32.4	16.7
Total	13.6	2.393	8.91	18.29		54.1	27.7	10.1

Abbreviations: lMR, Lymphocyte to monocyte ratio.

Bold indicates significance level at *p* < 0.05.

### 3.3 Univariate regression analysis for overall survival in cholangiocarcinoma patients

**FIGURE 2:**
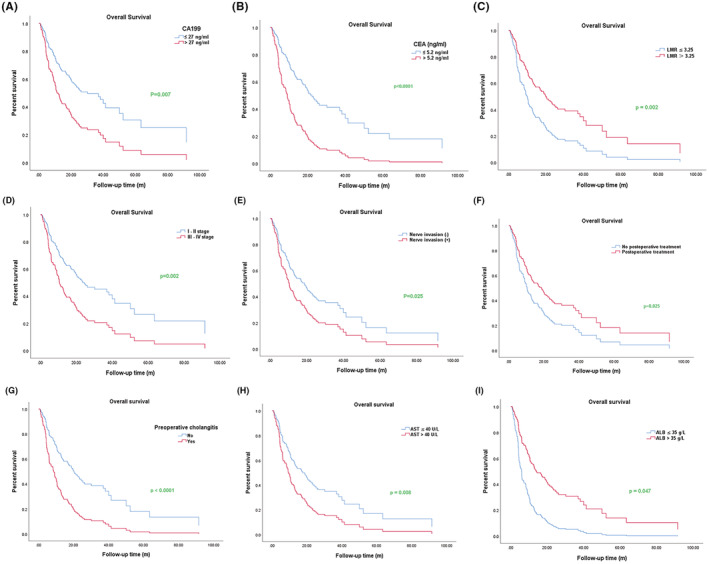
Kaplan–Meier survival curves of univariate regression analysis for overall survival (OS) in cholangiocarcinoma patients. (A) Kaplan–Meier survival curve for OS in carbohydrate antigen 19–9 (CA199) normal and elevated level groups. (B) Kaplan–Meier survival curve for OS in carcino‐embryonic antigen (CEA) normal and elevated level groups. (C) Kaplan–Meier survival curve for OS in low LMR (LMR≤3.225) and high LMR (LMR >3.225) groups. (D) Kaplan–Meier survival curve for OS in I‐II and III‐IV stage groups. (E) Kaplan–Meier survival curve for OS in nerve invasion and no nerve invasion groups. (F) Kaplan–Meier survival curve for OS in postoperative treatment and no postoperative treatment groups. (G) Kaplan–Meier survival curve for OS in preoperative cholangitis and no preoperative cholangitis groups. (H) Kaplan–Meier survival curve for OS in glutamic oxalacetic transaminase normal and elevated level groups. (I) Kaplan–Meier survival curve for OS in albumin normal and decreased level groups. *p* < 0.05 was statistically significant.

In order to further the prognostic factor for OS of cholangiocarcinoma patients, we used the Cox univariate regression model to analyze the effects of clinical characteristics on OS, and results showed that the levels of CA199 and CEA, TNM stage, nerve invasion, postoperative treatment, preoperative cholangitis, pathological type, AST, ALB, and LMR all significantly affected the OS **(**Table 3 **)**. As shown in Figure 2, we found that the OS of patients with elevated levels of CA199, CEA, and AST were shorter than patients with normal levels; however, there was a longer OS in the LMR high group than in the LMR low group. We also found that preoperative cholangitis, low albumin expression, staging (III‐IV), and nerve invasion were obviously associated with the shortened OS, while postoperative treatment was related to the longer OS.

**TABLE 3:** Univariate analysis for overall survival in cholangiocarcinoma patients.

Variable	Number	Relative risk	95.0% Confidence interval		*p‐*Value
			Inferior limit	Upper limit	
Age (year)					
≤55	48	1			
>55	97	0.782	0.511	1.197	0.257
Sex					
Male	78	1			
Female	67	0.778	0.524	1.154	0.212
HBV infection					
No	103	1			
Yes	42	1.212	0.772	1.901	0.403
CA199 (ng/mL)					
<27	35	1			
≥27	110	2.053	1.214	3.471	**0.007**
AFP (ng/mL)					
<10	126	1			
≥10	19	1.399	0.793	2.470	0.246
CEA (ng/mL)					
<5.2	84	1			
≥5.2	61	2.630	1.731	3.996	**<0.0001**
TNM stage					
I + II	56	1			
III+IV	89	1.987	1.280	3.084	**0.002**
Nerve invasion					
No	94	1			
Yes	51	1.611	1.062	2.445	**0.025**
Surgical margin					
No	136	1			
Yes	9	1.695	0.852	3.372	0.133
Lymphatic metastasis					
No	116	1			
Yes	39	0.874	0.568	1.343	0.539
Bile duct infringement					
No	124	1			
Yes	21	0.934	0.520	1.677	0.819
Vascular invasion					
No	55	1			
Yes	90	1.196	0.786	1.819	0.404
Tumor diameter (cm)					
≤2	22	1			
>2	123	0.849	0.480	1.500	0.573
Tumor number					
≤1	127	1			
>1	18	1.213	0.645	2.279	0.549
Satellite focal					
No	120	1			
Yes	25	1.100	0.651	1.857	0.722
Preoperative treatment					
No	19	1			
Yes	126	1.183	0.614	2.280	0.615
Postoperative treatment					
No	60	1			
Yes	85	0.633	0.425	0.944	**0.025**
Pathological differentiation					
high	10	1			0.827
moderately	72	0.926	0.423	2.026	0.847
poorly	63	0.878	0.582	1.326	0.538
Tumor location					
Left	50	1			0.613
Right	80	0.975	0.494	1.923	0.941
porta hepatis	15	0.796	0.394	1.605	0.523
Preoperative cholangitis					
No	95	1			
Yes	50	2.361	1.550	3.595	**<0.0001**
Pathological type					
ICC	89	1			
EHCC	56	1.591	1.065	2.378	**0.023**
ALT					
≤50	112	1			
>50	33	1.322	0.837	2.089	0.232
AST					
≤40	107	1			
>40	38	1.780	1.161	2.729	**0.008**
ALB					
≤35	6	1			
>35	139	0.401	0.162	0.990	**0.047**
TBIL					
≤26	132	1			
>26	13	1.781	0.947	3.348	0.073
LMR					
≤3.25	61	1			
>3.25	84	0.520	0.347	0.779	**0.002**

Abbreviations: ALB, albumin; AFP, alpha fetal protein; ALT, glutamic‐pyruvic transaminase; AST, glutamic oxalacetic transaminase; CA199, carbohydrate antigen 19–9; CEA, carcino‐embryonic antigen; EHCC, extrahepatic cholangiocarcinoma; ICC, intrahepatic cholangiocarcinoma; LMR, lymphocyte to monocyte ratio; TBIL, total bilirubin.

Bold indicates significance level at *p* < 0.05.

### 3.4 Multiple regression analysis for overall survival in cholangiocarcinoma patients

**FIGURE 3:**
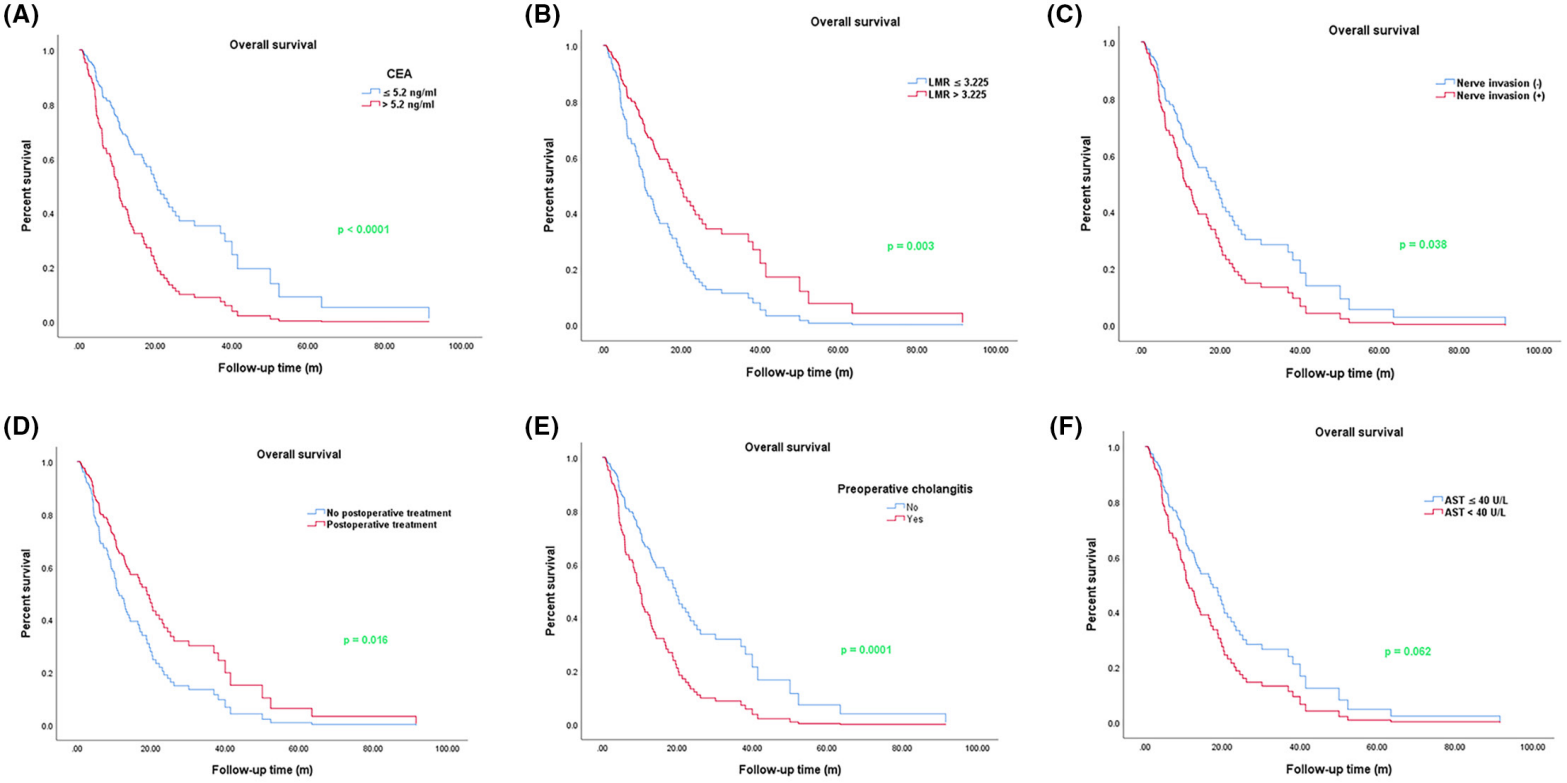
Kaplan–Meier survival curves of multiple regression analysis for overall survival (OS) in cholangiocarcinoma patients. (A) Kaplan–Meier survival curve for OS in carcino‐embryonic antigen (CEA) normal and elevated level groups. (B) Kaplan–Meier survival curve for OS in low lymphocyte to monocyte ratio (LMR) (LMR ≤3.225) and high LMR (LMR >3.225) groups. (C) Kaplan–Meier survival curve for OS in nerve invasion and no nerve invasion groups. (D) Kaplan–Meier survival curve for OS in postoperative treatment and no postoperative treatment groups. (E) Kaplan–Meier survival curve for OS in preoperative cholangitis and no preoperative cholangitis groups. (F) Kaplan–Meier survival curve for OS in glutamic oxalacetic transaminase normal and elevated level groups. *p* < 0.05 was statistically significant.

To further illustrate the influencing factors for OS of cholangiocarcinoma patients, we used the Cox multiple regression model to analyze the significant index that was statistically significant in univariate regression model analysis. As shown in Table 4, we found that preoperative cholangitis (*p* = 0.001, HR = 2.128, 95% CI (1.371–3.301)), elevated CEA level (*p* < 0.0001, HR = 2.313, 95% CI (1.488–3.594)), and nerve invasion (*p* = 0.038, HR = 1.596, 95% CI (1.027–2.481)) were risk factors for the OS of cholangiocarcinoma patients, while the high LMR level (*p* = 0.003, HR = 0.517, 95% CI (0.336–0.796)) and postoperative treatment (*p* = 0.009, HR = 0.582, 95% CI (0.387–0.875)) were protective factors for the OS of cholangiocarcinoma patients. As shown in Figure 3, the results of multiple regression analysis also indicated that the OS of patients with elevated level CEA were shorter than patients with normal level; however, there was a longer OS in the LMR high group than in the LMR low group. We also found that nerve invasion was obviously associated with the shorter OS, while postoperative treatment was related to the longer OS.

**TABLE 4:** Multivariate analysis for overall survival in cholangiocarcinoma patients

Variable	Number	Relative risk	95.0% Confidence interval		*P* value
			Inferior limit	Upper limit	
TNM stage					
I + II	56	1			
III+IV	89	1.109	0.692	1.779	0.666
Postoperative treatment					
No	60	1			
Yes	85	0.601	0.398	0.908	**0.016**
CA199 (ng/mL)					
<27	35	1			
≥27	110	1.132	0.635	2.020	0.674
CEA (ng/m)					
<5.2	84	1			
≥5.2	61	2.313	1.488	3.594	**<0.0001**
Nerve invasion					
No	94	1			
Yes	51	1.596	1.027	2.481	**0.038**
Preoperative cholangitis					
No	95	1			
Yes	50	2.128	1.371	3.301	**0.001**
AST					
≤40	107	1			
>40	38	1.523	0.979	2.369	0.062
ALB					
≤35	6	1			
>35	139	0.758	0.278	2.066	0.588
LMR					
LMR ≤3.225	61	1			
LMR >3.225	84	0.517	0.336	0.796	**0.003**

Abbreviations: ALB, albumin; AFP, alpha fetal protein; AST, glutamic oxalacetic transaminase; CA199, carbohydrate antigen 19–9; CEA, carcino‐embryonic antigen; LMR, lymphocyte to monocyte ratio.

Bold indicates significance level at *p* < 0.05.

### 3.5 Creation and use of the nomogram

**FIGURE 4:**
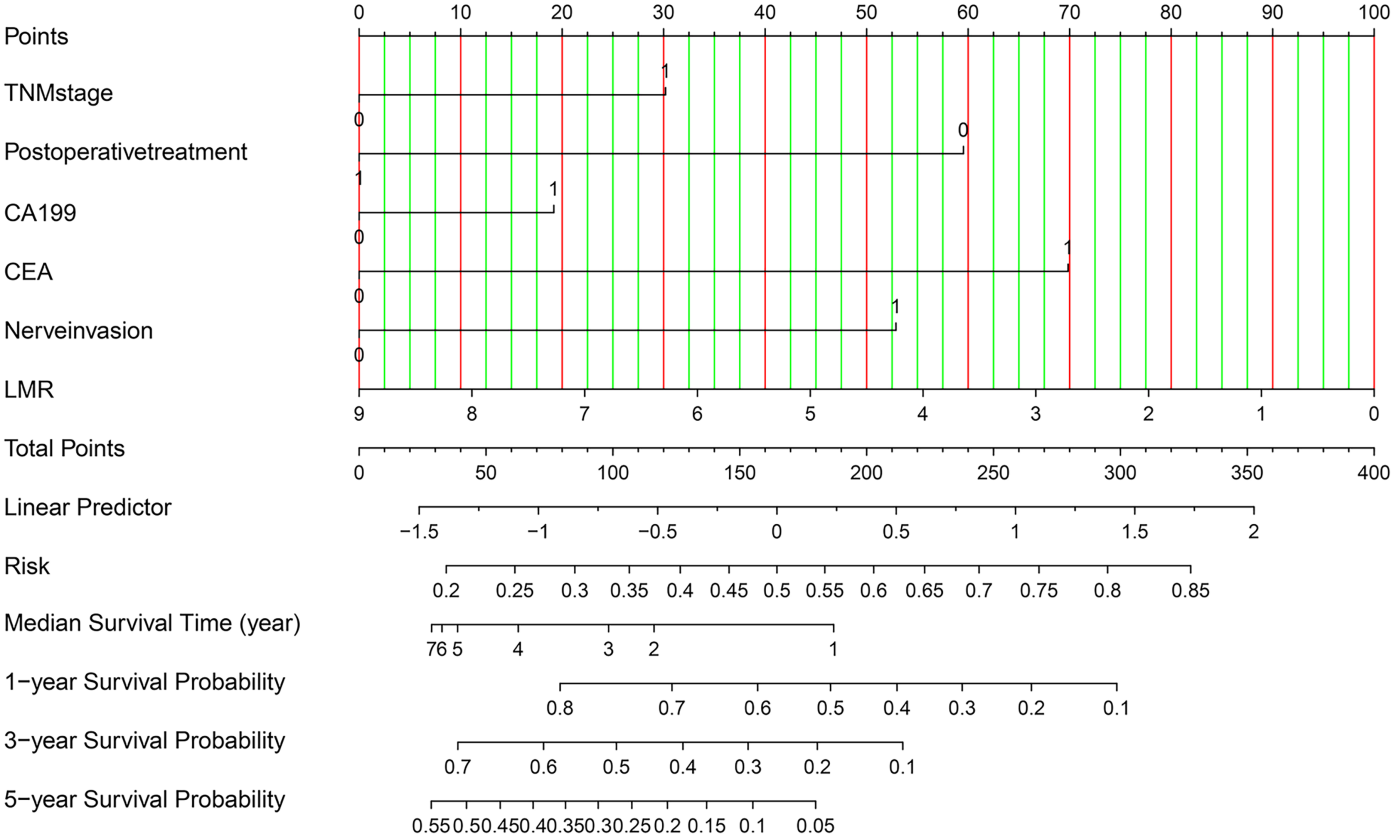
A nomogram for predicting median survival time and survival probability in cholangiocarcinoma patients. To use the nomogram, the value of each predictor is determined by drawing a line straight up to the point reference line. The points are summed, and a line is drawn downward from the total points line to find the predicted probability of node positivity (Postoperative treatment = 1, no postoperative treatment = 0; T1/2 = 0, T3/4 = 1; carbohydrate antigen 19–9 (CA199) <27 ng/mL = 0, CA199 ≥ 27 ng/mL = 1; carcino‐embryonic antigen (CEA) <5.2 ng/mL = 0, CEA ≥5.2 ng/mL = 1; nerve invasion = 0, no nerve invasion = 1).

In order to further evaluate the factors that significantly influenced the overall survival of CCA patients in the multivariate regression model, we constructed a Nomogram diagram to show the risk coefficient of some indexes, including TNM stage, the preoperative level of CA199 and CEA, nerve invasion, and the level of LMR. This compiled nomogram allowed for the estimation of the risk of OS and the prediction of median survival time and survival probability for each CCA patient **(**Figure 4 **)**. The steps to use the Nomogram are as follows: (1) determine the patient's value for each predictor, (2) draw a line up from each predictor to the vertex reference line, (3) add the points from each predictor, (4) locate the sum at the total point reference line, (5) draw a line down from the total point line, the median survival probability and 1‐, 3‐, 5‐year survival rates were obtained.

## 4 DISCUSSION

Cholangiocarcinoma (CCA) is a malignant tumor with a very poor prognosis. In many countries, the incidence, recurrence, and mortality rates of CCA remain high. Surgery is the only effective method; However, postoperative recurrence and metastasis significantly affect the prognosis of CCA patients. In addition, there is still a lack of effective markers to predict the prognosis of patients after surgery, which further provides direction for the treatment of patients after surgery. Previous studies exhibited that the OS and DFS (disease‐free survival) of CCA patients with low preoperative PLR (platelet to lymphocyte ratio) were significantly shorter than those of the high PLR group, indicating that the low level of PLR was associated with poor prognosis.^16^ In this study, we found that a new indicator, LMR, served as a prognostic biomarker for CCA patients with hepatic resection.

More and more studies have demonstrated that inflammatory indexes in peripheral blood and proinflammatory factors and chemokines in the immune microenvironment have significant effects on the prognosis of tumor patients.^17^, ^18^, ^19^ The inflammatory cells filling in tumor microenvironment are indispensable in tumor initiation, angiogenesis, invasion, and migration. Inflammatory cells and tumor cells used epithelial‐to‐mesenchymal transition (EMT) as a bridge to interact with each other at multiple levels and continuously, thus affecting the survival and prognosis of tumor patients.^20^ Various markers of systemic inflammation, including cytokines, C‐reactive protein, and monocyte, neutrophil, or lymphocyte count, as well as their ratios such as NLR (neutrophil/lymphocyte)^21^ and LCR (lymphocyte/C‐reactive protein),^22^ have been investigated for their prognostic roles in certain tumor populations. Furthermore, LMR, a biomarker of tumor inflammation and host immunity, was also associated with decreased mortality in tumors. As we all know, lymphocyte was a vital biomarker of host immune status, and lymphocytopenia was associated with lower survival rates for various cancers.^23^, ^24^ Monocyte was considered as surrogate markers of the tumor microenvironment. Recent studies have been demonstrated that LMR could serve as an effective marker for predicting the response of treatment and prognosis in lymphoma^25^ and tongue cancer patients.^26^ LMR could also predict survival in patients with advanced esophageal cancer who receive concurrent chemoradiotherapy, and low levels of LMR were significantly associated with poor prognosis in patients with esophageal cancer.^27^ In the present study, we found that LMR was also a prognostic marker that can be used to predict the overall survival of postoperative patients with CCA. The results of our study showed that the high LMR level (*p* = 0.023, HR = 0.609, 95% CI (0.397–0.935)) was a protective factor for the OS of CCA patients, and the OS of the low LMR group (10 months, 95% CI 6.766–13.234) was markedly lower (*p* = 0.001) than the high LMR group (20.23 months, 95% CI 14.706–25.754). We further employed the chi‐square test to analyze the correlation between LMR and clinical characteristics, and the results showed that the level of CA199 and CEA, TNM stage, and tumor number as statistically significant factors related to the LMR of CCA patients. Meanwhile, multivariate analysis of results indicated that the preoperative level of LMR and CEA, nerve invasion, and postoperative treatment independent prognostic factors for patients with CCA. Form our results in the previous part, we found that the preoperative level of LMR and CEA both predicted the OS of CCA patients with hepatic resection, and it was of great significance to judge the prognosis of patients with CCA in advance and to positively intervene in early treatment. Whether there was nerve invasion indicated by postoperative pathology was also a key factor affecting prognosis, and timely and postoperative treatment could also prolong the overall survival. Furthermore, unlike previous studies, we firstly created a nomogram figure to show the contribution of significant variables for overall survival in multivariate regression analysis, and to predict median survival time and 1‐, 3‐, and 5‐year survival rates for patients with cholangiocarcinoma.

The mechanism of decreased LMR in preoperative patients and poor prognosis was still unclear, of which the association of LMR with inflammation in the tumor microenvironment might be significant. A plausible explanation for this phenomenon was that the decreased LMR may reflect impaired immune function, as it was associated with lymphocytopenia and monocyte proliferation. Previous studies have shown that preoperative lymphocyte count was a good indicator of tumor prognosis and played a key role in the host's cytotoxic immune response to tumor, which was considered to reflect the general state of immune function, suggesting that lymphocyte‐mediated cytotoxicity could lead to the release of cytokines and inhibit the growth and metastasis of cancer cells.^28^ Lymphocytes could be divided into T cells, B cells, natural killer cells, etc., which played a key role in the anticancer reaction of the immune system by contributing to cancer cell apoptosis. Therefore, lymphopenia is supposed to be a surrogate signature of host immunological incompetence. Recent studies exhibited that monocyte has also been reported to be related to poor prognosis in various tumors, mainly because of tumor‐associated macrophages (TAMs).^29^ TAMs mainly came from circulating monocytes. There was increasing evidence that TAMs have major proto‐oncogenic activities, including promoting metastasis, immunosuppression, and tumor angiogenesis.^30^ Thus, it could be seen that lymphocytes and monocytes were involved in the anti‐cancer immune response and the formation of cancer microenvironment, thereby influencing the prognosis of CCA patients.

However, our study is only a single‐center retrospective study, and, due to a large number of patients with locally advanced stages in our included cases, our retrospective analysis of overall patient survival was lower than the data reported in previous studies. The reasons that tumor factors such as lymphatic metastasis and tumor diameter are not commonly reported as prognostic factors in this study are as follows: on the one hand, the sample may be small; on the other hand, confounding factors may interfere with the results. For example, patients with large‐diameter tumors did not have lymph node metastases; Patients with small tumor diameters existed in metastasis. In addition, although there was a significant difference in OS among patients with cholangiocarcinoma in the low and low LMR group, the AUC value (AUC = 0.599, 95% CI 0.498–0.700, *p* = 0.056) in our study was not high and there was no convincing result. Therefore, our result was only a preliminary study, and we need further study to demonstrate the effects of LMR on the OS of patients with cholangiocarcinoma. In the future, we still need the participation of multiple centers to further expand the sample size for verifying our conclusions. At the same time, more basic experiments including cell culture and animal model construction are needed to verify our findings at multiple levels.

In summary, this study was the first retrospective analysis of the correlation between preoperative LMR and OS in CCA patients. We found that there was a longer OS in the CCA patients with high LMR and normal CEA level, no nerve invasion, and postoperative treatment. We firstly created a nomogram plot to exhibit the contribution of significant variables for OS in multivariate regression analysis, and to predict survival time and rate, thereby providing a new sight for CCA patients with hepatic resection.

## AUTHOR CONTRIBUTIONS

**Minghe Lv:** Conceptualization (lead); data curation (lead); formal analysis (lead); investigation (lead); methodology (lead); software (lead); validation (lead); visualization (lead); writing – original draft (lead); writing – review and editing (lead). **Kun Wang:** Data curation (supporting); formal analysis (supporting); methodology (supporting). **Zhiyuan Zhang:** Formal analysis (supporting); validation (supporting). **Zhen Zhang:** Conceptualization (supporting); funding acquisition (equal); project administration (supporting); writing – review and editing (supporting). **Juefeng Wan:** Conceptualization (equal); funding acquisition (equal); project administration (lead); writing – review and editing (supporting).

## FUNDING INFORMATION

This work was supported by the Shanghai Anti‐Cancer Association (Grant Number: SACA‐AX201902) and Shenkang—Three‐year action plan clinical study (Grant Number: SHDC2020CR3082B).

## CONFLICT OF INTEREST STATEMENT

The authors declare that they have no competing interests.

## ETHICAL APPROVAL STATEMENT

The study was approved by the ethics committee of Fudan University Shanghai Cancer Center and the ethical code is 2003215‐1. Informed consent was obtained for all patients.

## Data Availability

Data sharing is not applicable to this article as no new data were created or analyzed in this study.
